# Quantitative Proteomics Using Formalin-fixed, Paraffin-embedded Biopsy Tissues in Inflammatory Disease

**DOI:** 10.35248/0974-276X.12.19.503

**Published:** 2019-10-03

**Authors:** Abhimanyu Amarnani, Joseph R. Capri, Puneet Souda, David A. Elashoff, Ivan A. Lopez, Julian P. Whitelegge, Ram R. Singh

**Affiliations:** 1 Department of Head and Neck Surgery, UCLA, Los Angeles, CA 90095, USA; 2 Department of Medicine/Rheumatology, UCLA, Los Angeles, CA 90095, USA; 3 The Pasarow Mass Spectrometry Laboratory, Semel Institute for Neuroscience and Human Behavior, UCLA, Los Angeles, CA 90024, USA; 4 Department of Medicine/Statistics Core, UCLA, Los Angeles, CA 90095, USA; 5 Department of Pathology and Laboratory Medicine, UCLA, Los Angeles, CA 90095, USA; 6 Molecular Toxicology Interdepartmental Program, UCLA, Los Angeles, CA 90095, USA; 7 Jonsson Comprehensive Cancer Center, David Geffen School of Medicine at UCLA, Los Angeles, CA 90095, USA

**Keywords:** Formalin-fixed paraffin-embedded, Fresh frozen, Inflammatory disease, Kidney biopsy, Lupus nephritis, Proteomics

## Abstract

**Background:**

Investigations in human disease pathogenesis have been hampered due to paucity of access to fresh-frozen tissues (FFT) for use in global, data-driven methodologies. As an alternative, formalin-fixed, paraffin-embedded (FFPE) tissues are readily available in pathology banks. However, the use of formalin for fixation can lead to the loss of proteins that appear during inflammation, thus introducing an inherent sample bias. To address this, we compared FF and FFPE tissue proteomics to determine whether FFPE-tissue can be used effectively in inflammatory diseases.

**Methods:**

Adjacent kidney slices from lupus nephritic mice were processed as FFPE or FFTs. Their tissue lysates were run together using proteomics workflow involving filter-aided sample preparation, in-solution dimethyl isotope labeling, StageTip fractionation, and nano-LC MS/MS through an Orbitrap XL MS.

**Results:**

We report a >97% concordance in protein identification between adjacent FFPE and FFTs in murine lupus nephritic kidneys. Specifically, proteins representing pathways, namely, ‘systemic lupus erythematosus’, ‘interferon-α’, ‘TGF-β’, and ‘extracellular matrix’, were reproducibly quantified between FFPE and FFTs. However, 12%−29% proteins were quantified differently in FFPE compared to FFTs, but the differences were consistent across experiments. In particular, certain proteins represented in pathways, including ‘inflammatory response’ and ‘innate immune system’ were quantified less in FFPE than in FFTs. In a pilot study of human FFPE tissues, we identified proteins relevant to pathogenesis in lupus nephritic kidney biopsies compared to control kidneys.

**Conclusion:**

This is the first report of lupus nephritis kidney proteomics using FFPE tissue. We concluded that archived FFPE tissues can be reliably used for proteomic analyses in inflammatory diseases, with a caveat that certain proteins related to immunity and inflammation may be quantified less in FFPE than in FFTs.

## INTRODUCTION

Fresh frozen tissue (FFT), when available, is the gold standard for clinical proteomics. However, for many chronic progressive diseases such as lupus nephritis (LN), this is unattainable, because FFTs must come from invasive biopsies with minimal tissue availability. Consequently, the proteome of LN has been evaluated in a small number of fresh-frozen kidney biopsies [1,2], which does not cover the full spectrum of LN. Advances in mass spectrometry (MS), especially improved protein digestion and direct quantification techniques, have made proteome analyses feasible for complex tissues. These advances include the ability to access the proteome in formalin-fixed, paraffin-embedded (FFPE) tissues that are a readily available treasure trove of information that can be harnessed via hospital tissue banks. Proteomics studies using FFPE tissues are being applied to kidney diseases including diabetic nephropathy [3] and renal carcinoma [4]. However, since the use of formalin for fixation crosslinks certain amino acids and the process of paraffin embedding might result in the loss of proteins, there is a possibility of introducing an inherent sample bias to proteomics studies using FFPE tissue [5]. Although, proteomic studies have been conducted using FFPE tissues, studies comparing FFPE and FFTs in inflammatory diseases or evaluating specifically for inflammatory pathways are lacking. We posit that proteins implicated in inflammation may be quantified less in FFPE comparted to FFTs. Hence, we conducted a quantitative MS-based proteomics workflow and data analytics platform to directly compare FFT and FFPE samples from LN kidneys.

## MATERIALS AND METHODS

### Tissue procurement and processing

To overcome the need for large human tissues, we used kidneys from NZM.2328 mice that develop glomerulonephritis that mimics LN in humans [6]. 10-month-old female NZM.2328 mice with proteinuria were perfused with ice-cold PBS during euthanasia. Kidneys were harvested, their two-halves processed as FFPE and FFTs, and contiguous 5μm sections obtained as mirrored regions (Supplemental Figure S1). Tissue sections were stored for 2–6 weeks prior to paraffin removal and protein isolation. Paraffin was removed and formalin fixation crosslinks reversed through successive incubations in xylene and ethanol. Animal experiments were performed according to the approved institutional protocol. Human FFPE kidney biopsies were obtained from UCLA Pathology Core, and processed, as described in Supplementary Figure S2.

### Protein isolation and FASP for digestion and peptide isolation

Using a previous protocol [7], FFPE and FFT sections were homogenized with Tris-HCL, dithiotreitol, and sodium dodecyl sulfate solution (pH 8.0), and incubated at 99°c with agitation. For FASP digestion, lysates with 150μg protein were placed atop a 30 kDa filter (Millipore Microcon YM-30) [8]. Consecutive urea washes removed contaminants and proteins were digested overnight, followed by washes into a new clean collection tube via NaCl and triethyl-ammonium bicarbonate. Isolated peptides were acidified and dried before isotope labeling.

### Peptide dimethyl isotope labeling and fractionation

Isolated peptides from FF and FFPE conditions were in-solution dimethyl isotope labeled, as described previously [9] (Supplementary Figure S3). Combined dimethyl labeled samples were fractionated via a modified StageTip procedure [10], adapted to produce 10 strong cation exchange (SCX) fractions. An initial C18 and SCX filter was created inside of a pipette tip with Millipore Extraction Disks (3M Millipore) and 9 other C18 only pipette tips were created. After StageTip conditioning, sample peptides were loaded onto the C18/SCX pipette tip, and increasing cuts of ammonium acetate were used to produce 10 fractions with release of peptides into the remaining 9 C18 tips.

### Nano-liquid chromatography with tandem mass spectrometry (nLC-MS/MS) analysis

Nano LC-MS/MS with collision induced dissociation was performed on an Orbitrap XL (Thermo Fisher, Waltham, MA) integrated with an Eksigent nano-LC. A prepacked reverse-phase column (Acutech Scientific C18) with a dimension of 75 μm × 20 cm containing resin (Biobasic C18, 5-μm particle size, 300-Å pore size, Acutech Scientific, San Diego, CA) was used for peptide chromatography and subsequent CID analyses. ESI conditions using the nano-spray source (Thermo Fisher) for the Orbitrap were set as follows: capillary temperature 220°C, tube lens 110 V and spray voltage of 2.3 kV. The flow rate for reverse-phase chromatography is 0.5 μl/min for loading and 400 nl/min for analytical separation (buffer A: 0.1% formic acid, 3% ACN; buffer B: 0.1% formic acid, 100% ACN). Peptide resolution gradient: 0–40% buffer B over 180 min, then 0% buffer B for 20 min of equilibration. The Orbitrap was operated in data-dependent mode with a full precursor scan at high-resolution (60,000 full width at half maximum, at m/z 400) and 10 MS/MS experiments at low resolution on the linear trap while the full scan was completed. For CID, intensity threshold was 5000, with mass range 350–2000. Spectra were searched using MaxQuant11 in which results with p<0.01 (99% confidence interval) were considered significant and indicating identity.

### Protein identification and quantification

MS raw data files were processed with MaxQuant (version 1.4.0.8) [11], searched against the UniProt mouse (4/4/13) database, and screened against the UniProt mouse reverse sequence and MaxQuant’s provided contaminant mus musculus*.fasta file. Variable modifications: N-termini acetylation, and methionine oxidation; Fixed modification: Cysteine carbamidomethylation. Identification setting: trypsin proteolytic enzyme, maximum 2 missed cleavage, minimum peptide count of 2. Peptides were specified to have a minimum length of 7 amino acids and max charge state of 7; max FDR 0.01. Triplex quantitation was processed utilizing light, intermediate, and heavy dimethyl labels to peptide N-termini and lysine residues, a 2-minute time window for matching identical peptides between fraction runs. Proteins were only deemed quantifiable if at least 2 peptides were quantified and one of those peptides was unique for that protein. Ratios were defined through normalization of geometric means. An example of protein identification and quantification process is illustrated in Supplemental Figure S3.

### Data analysis

Perseus (v1.5.2.3) [12] was used for protein identification overlap analysis, protein gene ontology classification, quantitative visualization, hierarchical clustering, and profile plots. Pearson correlations were calculated though the default ‘multiscatter’ graphical function in Perseus. Protein quantification was calculated as a ratio of dimethyl isotope labeling. Categorical annotations were accessed via the Perseus annotation download feature, based on majority protein ID and Uniprot. Pathway analysis was completed through Enrichr [13] to access gene ontology, KEGG, Wikipathways, Reactome, and GSEA databases. Hierarchical clustering was completed based on Euclidean distance, unsupervised with no k-means preprocessing. Venny was used for preparing venn diagrams (http://bioinfogp.cnb.csic.es/tools/venny/index.html). Graphpad prism was used to visualize pathway enrichment findings.

## RESULTS

### Correlation of protein intensities between FFT and FFPE tissues

**Figure 1:**
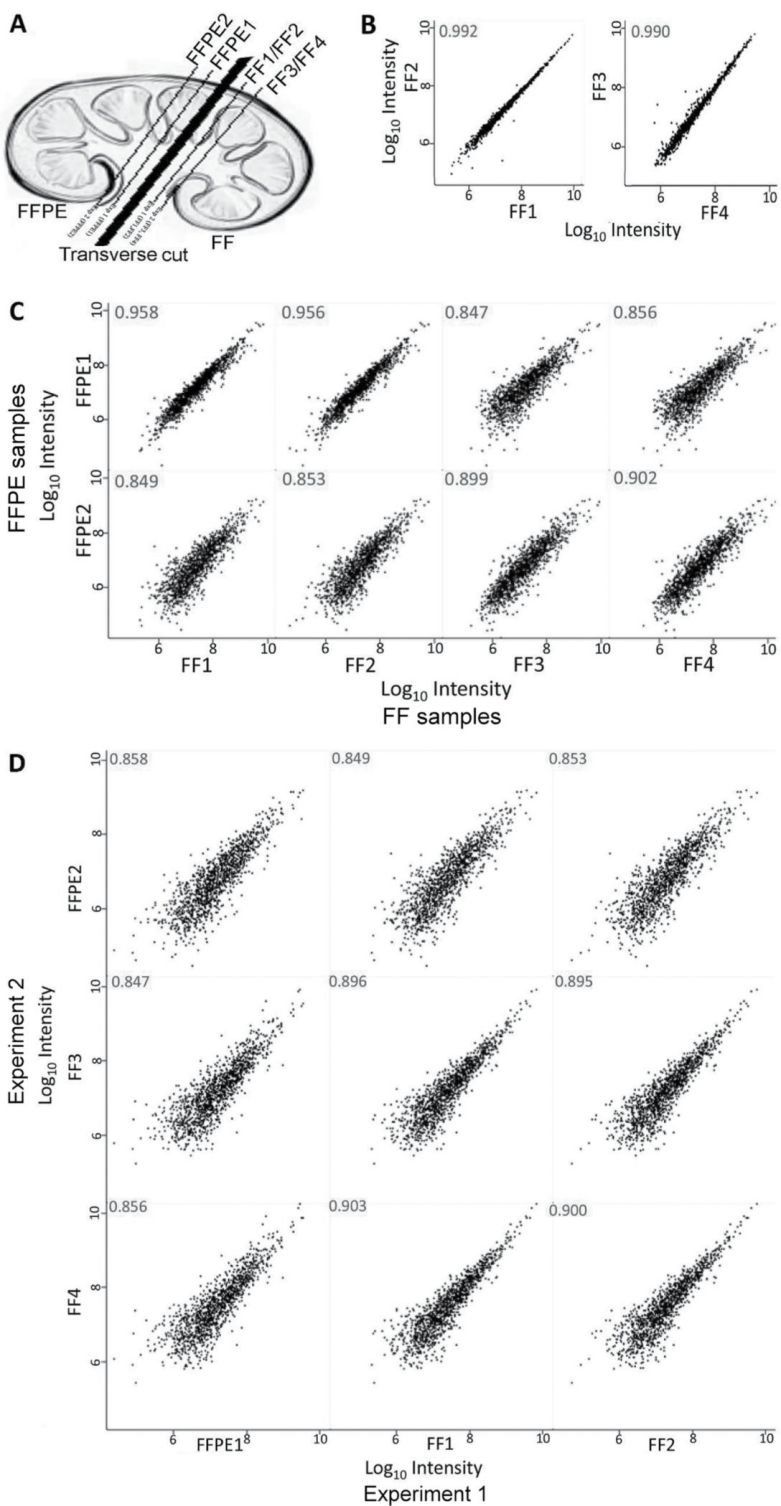
Scatter plots showing Pearson correlations of protein signal intensities between fresh frozen (FF) and formalin-fixed paraffin-embedded (FFPE) LN kidney tissues. Four slices from a nephritic lupus kidney were processed as FF and FFPE tissues (A) and run in two independent experiments: FFT protein lysate was run as two exact technical replicates (FF1 and FF2) and a contiguous FFPE slice lysate (FFPE1); the three lysates were labeled and run together (experiment 1). In experiment 2, two mirror image, but not contiguous, kidney slices from the same kidney were run as two exact technical replicates (FF3 and FF4), and a FFPE (FFPE2). (B) Correlation of exact technical replicates of FFT in experiments 1 (FF1 vs. FF2) and 2 (FF3 vs. FF4). (C) Correlations of FF (X-axis) vs. FFPE tissues (Y axis). (D) Correlation of protein intensities in experiment 1 (X axis) vs. experiment 2 (Y axis). Numbers on each panel represent correlation coefficient (R) values. Results shown are representative of three independent experiments, each using kidneys from different lupus-prone NZM.2328 mice.

To overcome the need for large human tissues, we used kidneys from NZM.2328 mice that develop glomerulonephritis that mimics LN in humans [6]. The adjacent kidney slices from these mice were processed as FFPE and FFTs (Figure 1A). Their tissue lysates were dimethyl isotope-labeled, and run together, as described in the Methods section. To establish our proteomics workflow’s variability, we split each FFT lysate into two, and ran these as exact technical replicates. As expected, their protein signal intensities were almost identical (Pearson’s correlation, R ≥0.99, Figure 1B). When comparing FFPE to FFTs (Figure 1C), R-value was 0.956–0.958 in contiguous sections, 0.899–0.902 in mirror image but not contiguous sections from the same kidney, and 0.847–0.856 across experiments. To further evaluate if this relatively lower correlation between FFPE and FFTs was due to a decreased concordance or due to experiment-to-experiment variations, we compared R-values across experiments (Figure 1D). Contiguous FFTs run in two separate experiments gave an R of 0.895–0.903, whereas contiguous FFPE samples run in two different experiments had an R of 0.858. Overall, these data show a strong comparability in protein intensities between contiguous FFPE and FFTs, with some variability in different kidney slices.

### Comparing protein identification between FFT and FFPE tissues

**Figure 2:**
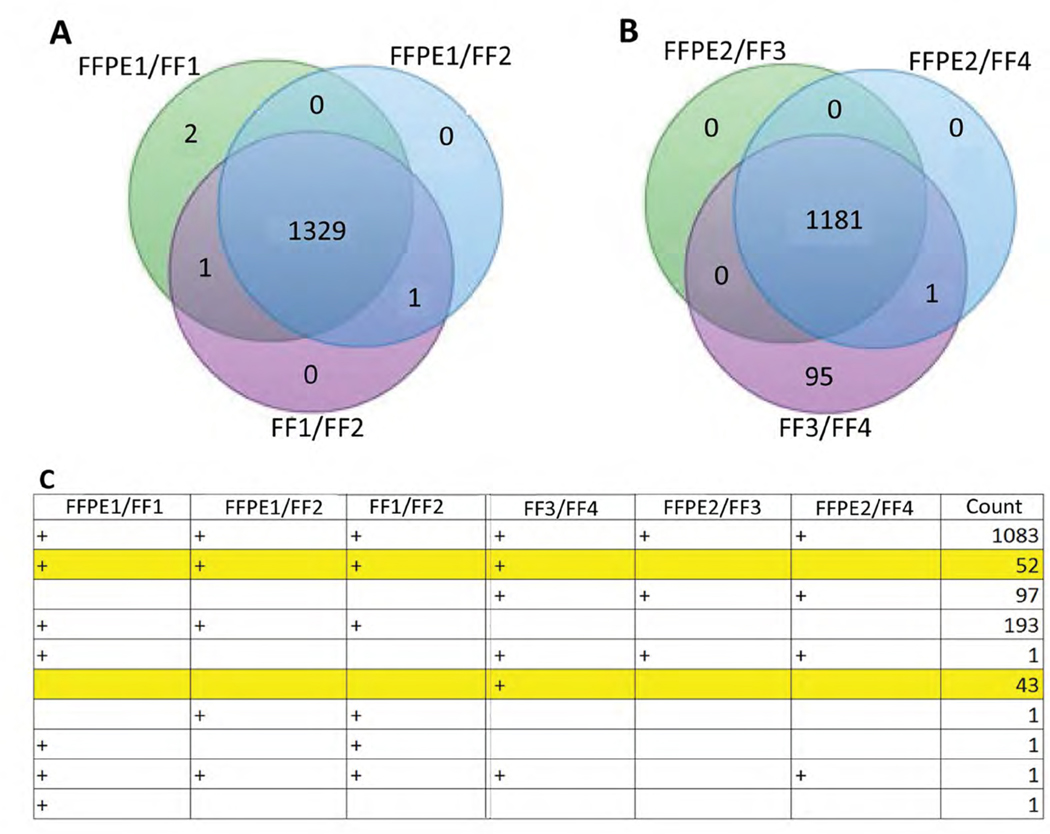
Comparison of protein identification between FF and FFPE processed LN kidney tissues. Four slices from a LN kidney were processed for proteomics analysis as in Figure 1A. Numbers of quantifiable proteins identified in FF and FFPE tissues are shown in contiguous (Experiment 1, (A) and non-contiguous, mirror-image kidney tissues (Experiment 2, (B, C) Number of proteins identified and quantifiable in each pairwise comparison. 1,083 proteins were detected in all conditions across separate experiments; 52 proteins were detected in contiguous sections (FF1/2 and FFPE1) in experiment 1, and contiguous sections (FF1/FF2 and FF3/FF4) run in two separate experiments, but not in a remote kidney slice from the same kidney (FFPE2); 97 and 193 proteins were detected exclusively in experiment 2 or experiment 1, respectively. The 43 proteins that were identified in FF3/FF4, but not in two FFPE tissues from the same kidney and in another FF tissue slice (FF1/FF2) from the same kidney included those in pathways for TNF signaling (VCAM1; LRP1), lysosome function (CD53; PSAP; AP3B1), RNA transport (CYFIP1; XPO1; EIF4G1), and drug metabolism (FMO2; UGT1A7C). There was not a single protein that was identified in both FF tissues but not in any FFPE tissue.

In a LN kidney, 1331 and 1333 proteins were quantifiable in contiguous FFPE and FFT slices, respectively, showing a concordance of 99.8% (experiment 1, Figure 2A). A lower concordance – 92.6% (1182/1277) – was seen in mirror image, but not contiguous, FFPE and FFTs (experiment 2, Figure 2B). There was some discrepancy in proteins identified between FFPE and FFTs. This could be due to some proteins not being identifiable in FFPE tissues or differences in tissue composition between heterogeneous FFPE and FFT slices. There was not a single protein that was identified in both FFTs, but not in any FFPE slices (Figure 2C).

### Comparing protein quantification between FFT and FFPE tissues

**Figure 3:**
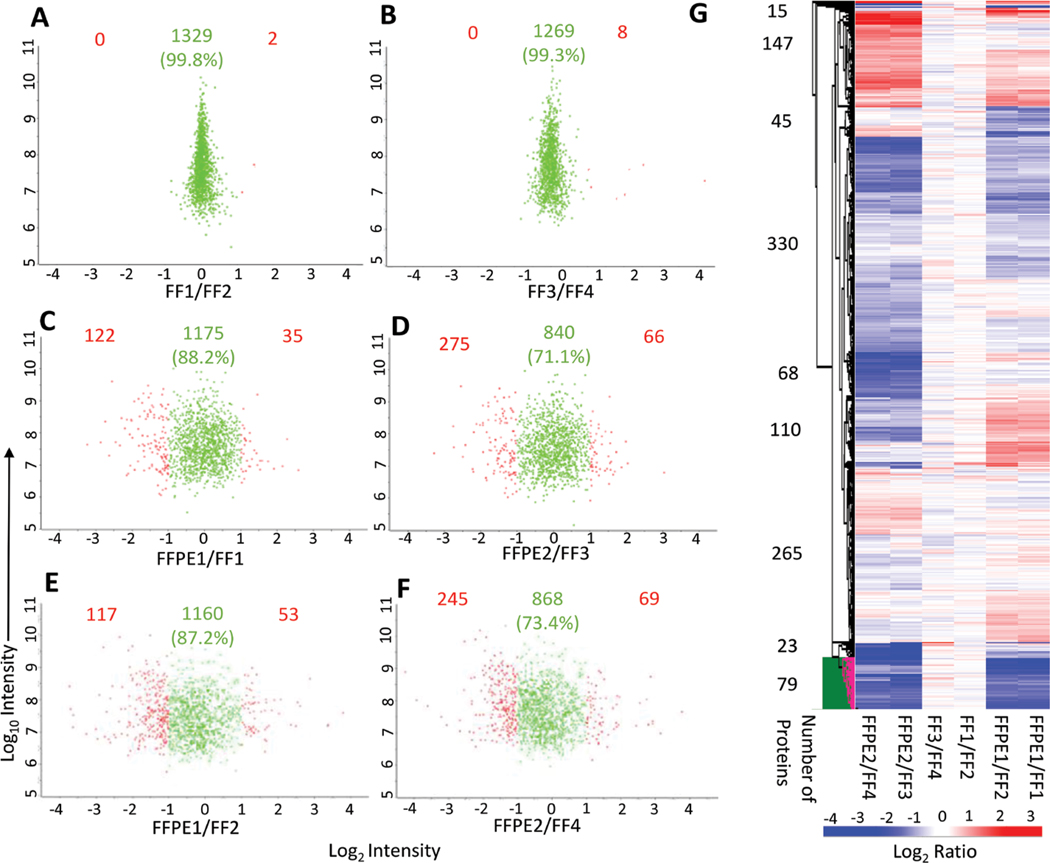
Pairwise comparisons of relative quantification ratios of proteins between FF and FFPE LN kidney tissues. (A, B) Quantitative comparison of exact technical replicates, showing that 99.8% and 99.3% of proteins were quantified within the range of −1 and +1 (green). Proteins that were quantified beyond the range of −1 to +1 are highlighted in red. (C-F) Comparison of FFPE and FF samples, showing differences in protein quantification. (G) Hierarchical clustering with no k-means preprocessing and average Euclidean distance linkage clustering. Note that relative to proteins quantified in FF tissues (FF1/FF2 and FF3/FF4), 147 proteins were most consistently increased and 79 proteins were most consistently lower in FFPE tissues across experiments.

**Figure 4:**
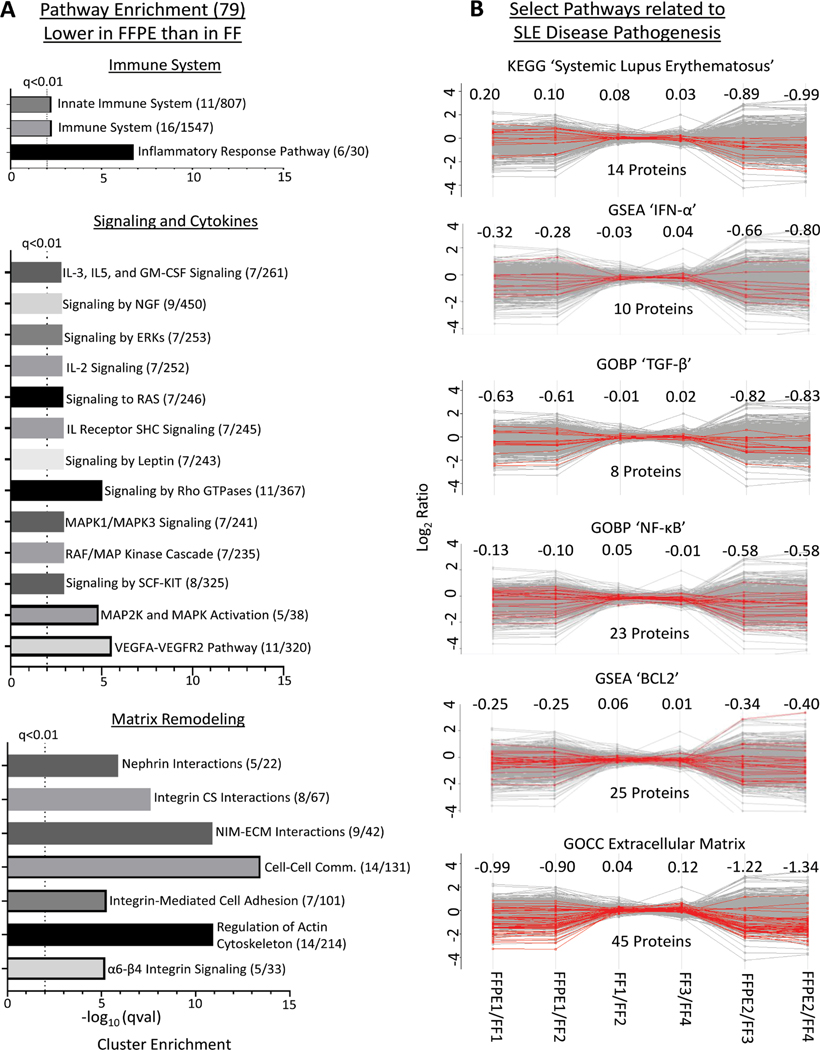
Reliability of FFPE samples for quantification of proteins relevant to biological processes and inflammatory disease pathology. (A) Characteristics of 75 proteins that were most decreased in FFPE relative to FF tissues. Pathway analysis of these proteins was performed using Enrichr [13] to access the KEGG, WikiPathways, and Reactome, revealing significant enrichment of pathways relevant to immune system function, intracellular signaling, cytokine functions, and matrix remodeling. Significantly enriched pathways shown have a Fisher’s exact test adjusted p value (qval) < 0.01. X axis indicates the –log(qVal) for each pathway. (B) Reliability of protein quantification in FFPE compared to FF tissues for pathways relevant to LN pathogenesis. Pathway analysis of proteins that were consistently quantified in all FFPE and FF samples was conducted for representative pathways indicated in each panel. The number of quantified proteins within each pathway are indicated below each graph. Numbers above each graph represent the average log_2_ ratio of the protein profiles highlighted in each pathway. Note that proteins included in the respective pathways shown were quantified in FFPE samples compared to FF on average within a range of −1 to 1, except for extracellular matrix proteins. Highlighted proteins (red lines) are those that are included in the indicated pathways, annotated within Perseus [12] through GOBP, GOCC, GSEA, and KEGG. A list of these proteins is provided in Supplementary Table S2. [Comm., communication; CS, cell surface; NIM-ECM, non-integrin membrane-extracellular matrix.]

We conducted pairwise comparisons of the relative quantification ratios of proteins identified in different samples. In exact technical replicates, 99.3%−99.8% of proteins were quantified within [−1 to +1] on a log_2_ scale (Figures 3A and 3B). Using this cut-off as a baseline variability of our workflow, we found that when comparing FFPE and FFTs, 87.2%−88.2% of proteins in experiment 1 (FFPE1 *vs.* FF1/FF2; Figures 3C and 3E) and 71.1%−73.4% in experiment 2 (FFPE2 *vs.* FF3/4; Figures 3D and 3F) were quantified within the baseline variability range. Hierarchical clustering further showed that quantitative differences – increase or decrease – in FFPE compared to FFTs were mostly consistent across separate experiments (Figure 3G). To determine whether proteins related to certain biological processes were quantified differently in FFPE tissues, we conducted pathway analyses of 79 proteins that were consistently most decreased in FFPE as, compared to FFTs, across both experiments (Figure 3G). These 79 proteins were enriched in pathways relevant to immune system function, intracellular signaling, cytokine functions, and matrix remodeling (Figure 4, Supplementary Table S1). Nevertheless, most proteins within pathways implicated in LN pathogenesis, namely SLE, IFN-α, TGF-β, NF-αB, and BCL2 were quantified similarly in FFPE and FFTs (Figure 4B, Supplementary Table S2). Some proteins were increased when comparing FFPE1 to FF1/2 but decreased between FFPE2 and FF3/4. This cluster of proteins was not enriched in pathways related to immune function or lupus pathogenesis, but included proteins associated with normal kidney tissues, particularly with kidney tubules. This suggests that differences in tissue composition between different sections from the same kidney may have contributed to some quantitative differences between different samples. Next, we compared the proteomes of FFT and FFPE LN kidney slices relative to healthy (BALB/c) kidneys (Supplemental Figure S4). The protein signal intensities between FFPE and FFT LN samples relative to BALB/c showed a strong correlation. A hierarchical clustering showed that the majority of proteins were quantified comparably between FFT and FFPE LN kidneys relative to BALB/c kidneys, suggesting that FFPE tissues can be reliably used to quantify proteins that are differentially expressed between LN and healthy kidneys.

### Pilot proteomics study using human FFPE kidney biopsies

**Figure 5:**
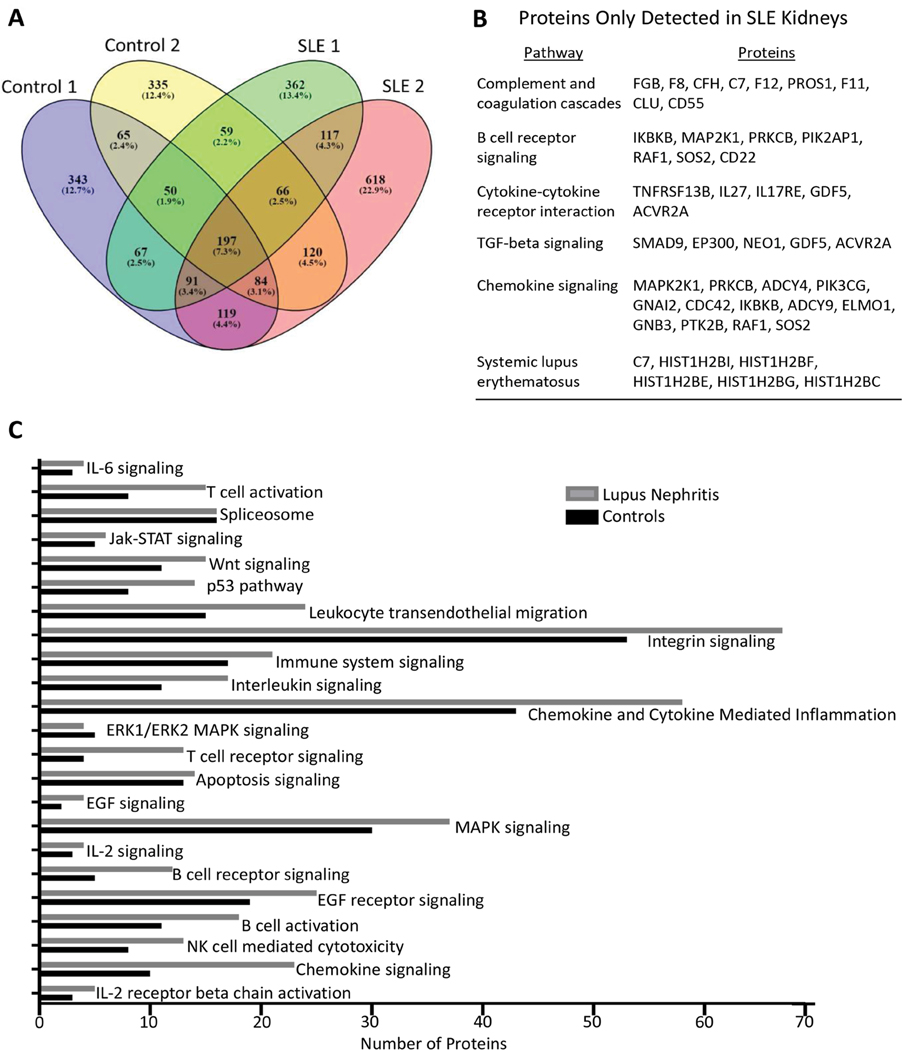
Pilot study using human FFPE kidney biopsies to compare the LN and normal kidney proteome. Kidney biopsy sections were obtained from two each of LN and control subjects, deparaffinized, and the extracted protein (3 μg) used to obtain a protein profile using NanoLC-MS/MS, as described in Supplementary Figure S1. (A) Venn diagram showing the distribution of high-confidence proteins as determined by the MASCOT software and SwissProt database. (B) Select proteins identified in SLE samples, but not in control samples. C) Numbers of proteins identified in SLE kidneys vs. control kidneys organized by selected immune activation and signaling pathways annotated through the DAVID functional annotation tool [22].

As a proof of principle, we conducted a pilot proteomics study using human FFPE kidney biopsy sections from LN and control subjects (Supplementary Figure S2). In two kidneys from patients with SLE, one each with mild mesangioproliferative and crescentic LN, we detected 1009 and 1412 high confidence hits (CI>95%), respectively, as compared to 1016 and 976 in two normal kidneys (Figure 5A). Of the high confidence hits, 117 proteins were found in both SLE kidneys, but not in either of the controls, whereas 65 proteins were found in both normal kidneys but not in either of the SLE kidneys. Proteins found only in SLE kidneys, but not in controls, mapped to pathways previously implicated in LN disease pathogenesis, including complement and coagulation cascades, B cell receptor signaling, cytokine-cytokine receptor interaction, TGF-◦ signaling, chemokine signaling, and SLE (Figure 5B). SLE kidneys also had a higher number of proteins related to select immune activation and signaling pathways than control kidneys (Figure 5C). These results suggest that FFPE tissue can be used to identify proteins that are differentially expressed in LN as compared to normal kidneys, including proteins that have been implicated in SLE pathogenesis.

## DISCUSSION

We demonstrate a strong comparability in the proteome between FFTs and FFPE-processed kidney slices from lupus nephritic mice. Over 1300 proteins were identified in both FFPE and FFTs, and 71.1%−88.2% of proteins were quantified within the log_2_ range of −1 and 1 when comparing FFPE vs. FFTs. These proteins mapped to pathways relevant to LN disease pathogenesis. However, 75 (7.3%) proteins were quantified less in FFPE compared to FFTs. Thus, some proteins may be under sampled in studies using FFPE tissues. Our study does not rule out the possibility that some quantitative differences between FFPE and FFTs might be due to differences in the composition of adjacent tissue slices. Nevertheless, a near complete concordance in proteins identified in contiguous FFT and FFPE kidney slices suggest that FFPE tissues can be reliably utilized for proteomics studies in inflammatory diseases such as LN. The present study is the first report, to our knowledge, of LN kidney proteomics using FFPE tissue. While we do find in the literature three reports of global proteomic analysis of renal tissue in LN, these used only fresh-frozen kidney biopsies from a small number of patients with one or two classes of LN [1,2,14]. While we did not find reports on LN kidney proteomics using FFPE tissue, we did find one proteomics study comparing FFT to FFPE kidney tissue. However, the FFPE to FFT comparison in this study was of hypertensive rat kidneys and the comparison occurred across separate experiments [15].

Therefore, we used a proteomics workflow involving filter-aided, sample preparation (FASP) [7,16,17], in-solution dimethyl isotope labeling [9], strong cation exchange StageTip fractionation [7,10,16], along with nano-LC MS/MS through an Orbitrap XL MS, which allowed for a direct comparison of different tissues in the same run. Building up on the murine data showing that FFT and FFPE kidney tissues provided comparable proteomics data in an inflammatory disease, we pilot tested the use of FFPE samples in human inflammatory diseases, we conducted a proof of principle study using FFPE kidney biopsies from patients with LN and controls. We found that more proteins representing pathways that have been implicated in SLE pathogenesis were detected in LN kidneys than in normal kidneys. These findings form the basis for comprehensive proteomics studies using a larger set of LN and control kidney biopsies. Elegant studies have assessed gene expression in LN using fresh-frozen kidney tissues [18]. However, a study to establish whether transcript levels of a given gene can be used as proxies for the corresponding protein levels found that the direct Pearson’s correlation between RNA and protein levels in normal kidney tissues was only 0.53 [19]. Consequently, our data demonstrating the utility of readily accessible FFPE tissues compared to limited availability of FFTs may have far-reaching implications for identifying proteins relevant to human disease pathogenesis. The initial proteomics studies using the whole tissue can then form the basis for future studies to perform proteomics studies using laser-capture microdissection of as few as 5,0000 FFPE cells [20] or by targeted proteomics analyses of frozen cell suspensions using mass-cytometry [21].

## CONCLUSION

In conclusion, the data described herein gives us confidence that large-scale proteomics studies using archived, FFPE-processed clinical tissues, which are readily available in hospital pathology banks, can provide insight into the pathogenesis of chronic inflammatory diseases, such as LN.

## Supplementary Material

Suppl fig

Suppl data

## Abbreviations:

BCL2: BCL2 Apoptosis Regulator
CI: Confidence Interval
FASP: Filter Aided Sample Preparation
FFPE: Formalin Fixed Paraffin Embedded
FF: Fresh Frozen
GOBP: Gene Ontology Biological Process
GOCC: Gene Ontology Cellular Component
GSEA: Gene Set Enrichment Analysis
IFN-α: Interferon Alpha
KEGG: Kyoto Encyclopedia of Genes and Genomes
LN: Lupus Nephritis
MS: Mass Spectrometry
NF-κB: Nuclear Factor Kappa-Light-Chain-Enhancer of Activated B Cells
NZM-2328: New Zealand Mixed 2328
nLC-MS/MS: nanoLiquid Chromatography- Tandem Mass Spectrometry
SLE: Systemic Lupus Erythematosus
TGF-ß: Transforming Growth Factor-Beta
