# Supplementary material for: Quantitative Proteomics Using Formalin-fixed, Paraffin-embedded Biopsy Tissues in Inflammatory Disease
Source: J Proteomics Bioinform. Author manuscript; Available in PMC 2020 May 19. (PMC7236785; doi:10.35248/0974-276X.12.19.503)
Supplement: Suppl fig [file NIHMS1065650-supplement-Suppl_fig.pdf]

SUPPLEMENTARY MATERIAL

CONTENTS

Figure S1. Experimental Design of the study.

Figure S2. Human kidney biopsies used in the study.

Figure S3. An example of protein identification and quantification process.

Figure S4. Quantifying proteins in fresh frozen (FF) vs. formalin-fixed, paraffin-embedded (FFPE) lupus nephritic kidney relative to healthy kidney tissues.

Table S1. The 79 proteins that were most decreased in formalin-fixed, paraffin-embedded (FFPE) samples, compared to fresh frozen (FF), across both experiments: provided as a separate excel sheet (Supplemental Table S1.xlsx).

Table S2. The proteins within the pathways illustrated in Figure 4B.

Supplementary References

SUPPLEMENTARY FIGURES

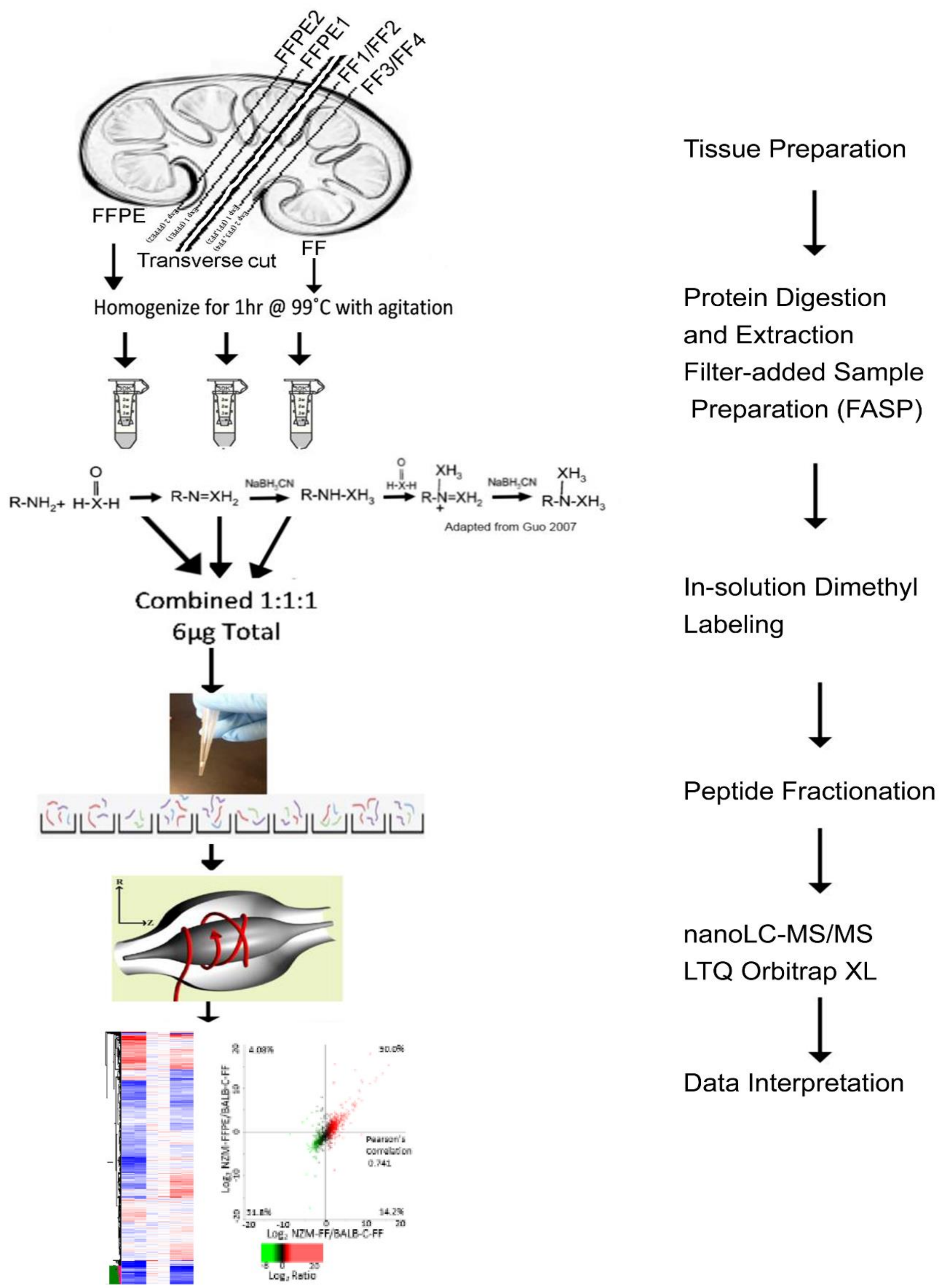

Supplemental Figure S1. Experimental Design of the study. Four slices, each of 5μm, from a lupus nephritic kidney were processed as fresh frozen (FF) and formalin-fixed, paraffin-embedded (FFPE) tissues, and run in two independent experiments: FFT protein lysate was run as two exact technical replicates (FF1 and FF2) and a contiguous FFPE slice lysate (FFPE1); the three lysates were labeled and run together (experiment 1). In experiment 2, two mirror image, but not contiguous, kidney slices from the same kidney were run as two exact technical replicates (FF3 and FF4), and a FFPE (FFPE2). Further steps included trypsin digestion, triplex dimethyl isotope labeling, and the combination of each condition's peptides into a single solution. Solutions were fractionated via a stage-tip method and analyzed with nLC-MS/MS.

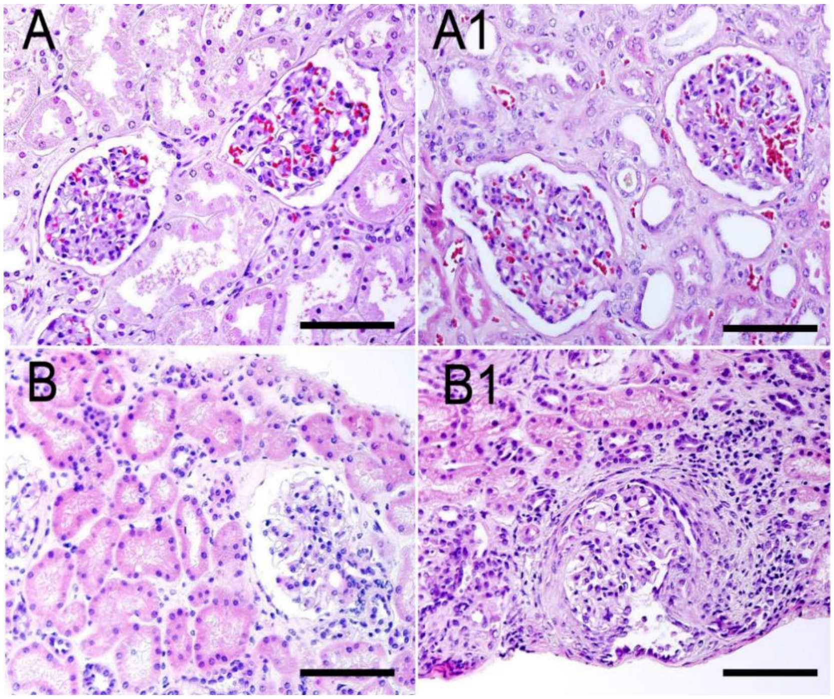

Supplementary Figure S2. Human kidney biopsies used in the study. H&E stained kidney biopsy sections from two each of SLE patients (lower panels, B and B1) and control subjects (upper panels, A and A1) and are shown. Control kidneys have normal appearing glomeruli and tubules. Kidney biopsies from SLE patients show mild mesangioproliferative LN with mesangial hypercellularity (B) and diffuse proliferative LN with marked glomerular proliferation and accentuated lobular architecture, karyorrhexis, crescent formation, and interstitial infiltration (B1). Four µm sections of these FFPE kidney biopsies were obtained from UCLA Pathology Core in accordance with the approved Institutional Review Board protocol. Tissues were deparafinnized with 100% xylene for 10 minutes, 100% ethylic alcohol, and rehydrated with descending ethylic alcohol 95% in water v/v, (1X), 80%(1X) and 50% (1X). Samples were prepared using the Liquid Tissue MS protein prep kit (Expression Pathology, Rockville, MD) following a previously described protocol [1]. Protein concentration in the sample was measured using the micro BSA protein assay method. The extracted tryptic peptide samples (3 µg) were fractionated on a HPLC column, and analyzed with a quadruple time-of-flight hybrid mass spectrometer (Applied Biosystems Q-STAR XL) with nanobore LC-MSMS capability and equipped with ESI, nanospray and APCI sources. We only identified proteins that were found in 95% confidence interval or higher in relation to the total ion score and strength as determined by the MASCOT software and SwissProt database. Automatic isotope correction was carried out by both software packages using the values supplied with the Applied Biosystems reagents. Then, the UniProt database was used to elucidate the biological process, cellular location and molecular function of each individual protein. Pathway mapping was conducted using the publicly available DAVID Functional Annotation and Bioinformatics Analysis Software [2].

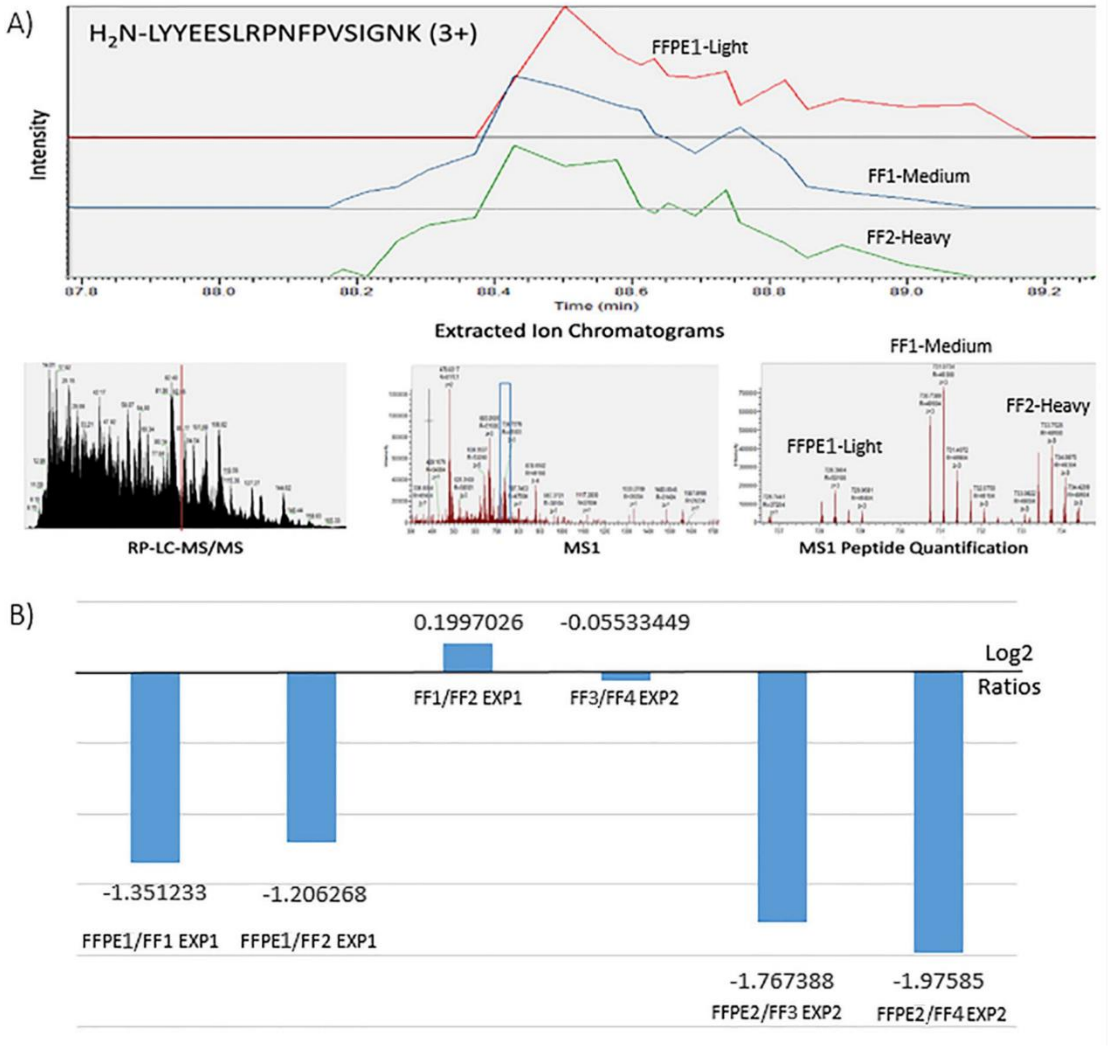

Supplementary Figure S3: **An example of protein identification and quantification process**.

A) In-solution dimethyl labeling and quantification for complement factor H. This shows the extracted ion chromatograms and the mass spectra from a triplex stable dimethyl isotope labeled peptide (H2N-LYYEESLRPNFPVSIGNK). This peptide was produced from trypsin cleavage and is specific to the complement factor H protein. Once differentially labeled, one FFPE condition as well as two technical replicate FF conditions were combined in a 1:1:1 ratio. This resulted in the simultaneous detection of each of the labeled peptide ion fragments. As noted under the MS1 peptide quantification window, an expected m/z shift increase was observed among the conditions. This allowed for the relative quantification of peptide between each pair of conditions via the area under the curve from the extracted ion chromatogram [3].

B) Example of quantification of complement factor H in each pair of conditions. The relative quantification of complement factor H peptide between each pair of conditions in both experiments is illustrated. These values were derived from a geometric mean of the quantification from each of its detected peptides, thereby providing quantification for the complement factor H protein. The quantification of all proteins analyzed was conducted via the Maxquant analysis and Perseus visualization system in a manner identical to this.

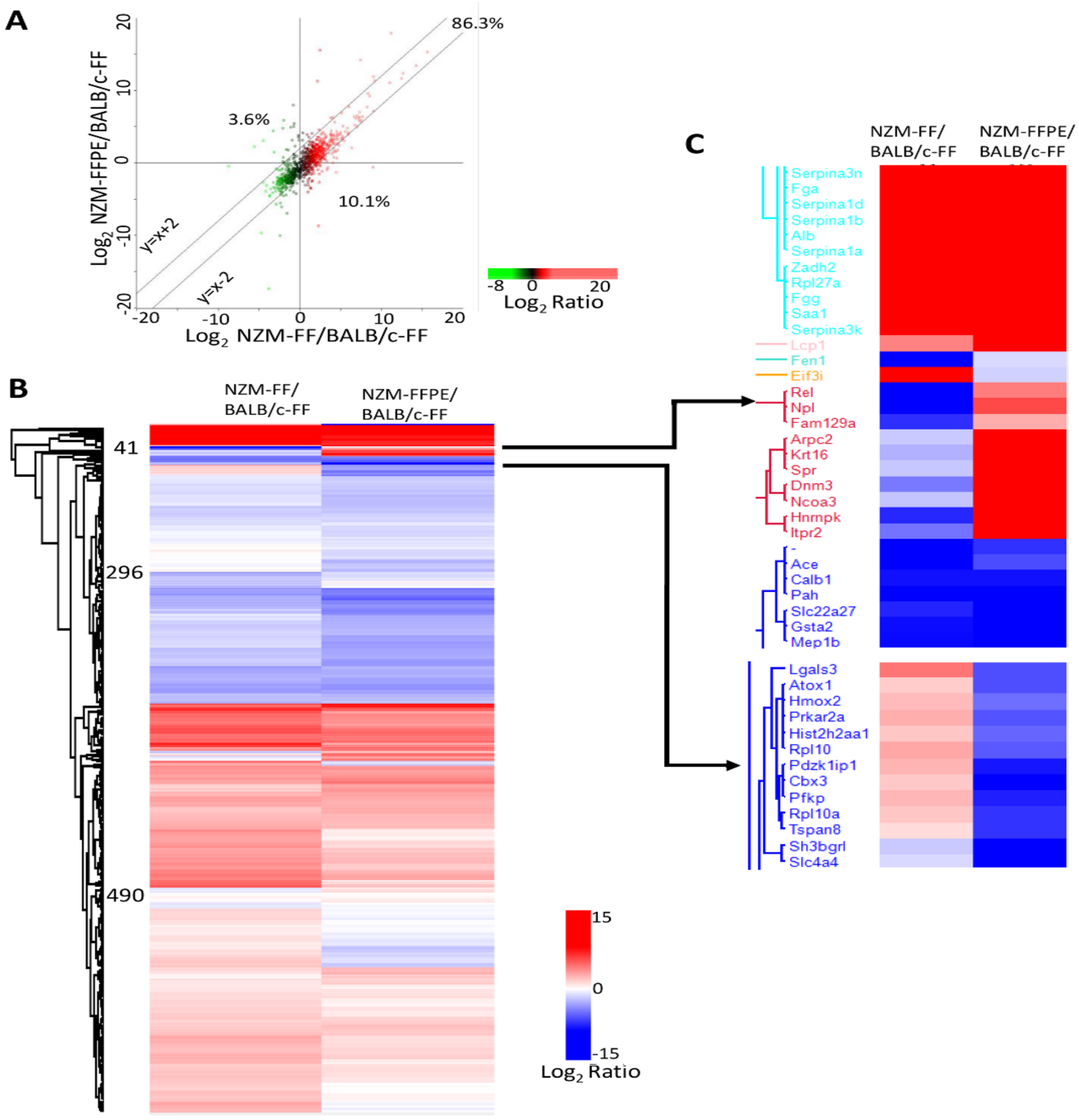

Supplementary Figure S4: Quantifying proteins in fresh frozen (FF) vs. formalin-fixed, paraffin-embedded (FFPE) lupus nephritic kidney relative to healthy kidney tissues. Kidney slices from 10-month-old lupus-prone NZM.2328 female mice with high-grade proteinuria were processed as FFPE and FF tissues. Kidney slices from 10-month-old healthy BALB/c female mice were processed as FF tissues. Proteins extracted from BALB/c-FF, NZM-FF, and NZM-FFPE tissues were dimethyl isotope labeled, and processed together for quantitative proteomics evaluation, as described in Methods. **A)** Comparison of protein signal intensities between NZM-FFPE/BALB/c and NZM-FF/BALB/c gave a Pearson correlation coefficient of 0.741. Note that 86.3% of proteins quantified were within the functions y=x+/-2, with 3.6% proteins above and 10.1% proteins below this level in FFPE as compared to FF tissues. **B)** Hierarchical clustering of proteins indicating that the majority of proteins were quantified comparably between NZM-FFPE/BALB/c and NZM-FF/BALB/c ratios. This included proteins relevant for inflammatory disease, including PPAR, TNF-alpha, NF-kappaB, PI3K-Akt, EGFR1, IL-3, and GL13 signaling pathways, metabolism, antigen processing and presentation, apoptosis, and matrix remodeling (Amarnani A, Singh RR, unpublished data). **C)** 10 proteins were quantified more and 12 proteins were quantified less in FFPE vs. FF tissue. The proteins that were quantified less include EIF3I, LGALS3, ATOX1, HMOX2, PRKAR2A, HIST2H2AA1, RPL10, PDZK1IP1, CBX3, PFKP, RPL10A, TSPAN8, SH3BGRL, and SLC4A4. The proteins that were quantified more include REL, NPL, FAM129A, ARPC2, KRT16, SPR, DNM3, NCOA3, HNRNPK, and ITPR2.

SUPPLEMENTARY TABLE

Supplementary Table S1: The 79 proteins that were most decreased in formalin-fixed, paraffin-embedded (FFPE) samples, compared to fresh frozen (FF), across both experiments. (Supplemental Table S1.xlsx)

Supplementary Table S2: The proteins within the pathways illustrated in Figure 4B.

**Pathways**	**Proteins**
KEGG [4] 'SLE'	ACTN1, H3FA, HIST1H2AL, C3, H2AFV, HIST1H2BP, H2-AA, SNRPB, ACTN4, SNRPD1, SNRPD3, HIST1H4A, H2AFY, H2AFY2
GSEA [5] 'IFN-alpha'	PLEC, CYC5, PSME1, B2M, CALR, UBE2D2B, PHB, LGALS3BP, DDX17, PDXK
GOBP [6] 'TGF-ß'	DAB2, GLG1, COL4A2, ITGB1, COL1A1, HSPA5, FERMT2, STRAP
GOBP [6] 'NF-κB'	CHP1, FLNA, TFG, EEF1D, ICAM1, HSPB1, LGALS1, GSTP1, PRDX3, TGM2, CAT, PRDX1, UBE2N, MTPN, RPS3, S100A13, CTNNB1, CLU, TMED4, PRDX2, CTH, RHOA, PSMA6
GSEA [5] 'BCL2'	AGPS, ITIH4, KNG1, CFH, AHCYL, GSTM4, IMPA1, IGHG1, FN1, HSPB1, HIST1H1C, MUT, ASS1, HSPA1A, GPX3, NEDD4, DBT, CALM1, GNAS, ANXA4, BPHL, SLC5A2, SLC25A11, GALK1, TAGLN2
GOCC [6] 'Extracellular Matrix'	HMCN2, COL15A1, AGRN, HSPG2, COL14A1, COL6A3, COL12A1, IGFP7, LAMB1, COL18A1, LAMC1, GLG1, TINAGL1, NID2, COL4A1, LAMC1, ANXA2, ALB, COL4A2, SOD1, ITGB1, NID1, COL1A1, FN1, APCS, CALR, LGALS1, LGALS3, CTSD, MFGE8, TGM2, BGN, VTN, LUM, COL6A1, COL6A2, CLU, LGALS3BP, LAMA5, LAMB2, ITGA6, TNC, EMILIN1, COL4A3, TINAG
GSEA [5] 'BCL2'	AGPS, ITIH4, KNG1, CFH, AHCYL2, GSTM5, IMPA1, IGHG1, FN1, HSPB1, HIST1H1C, MUT, ASS1, HSPA1A, GPX3, NEDD4, DBT, CALM1, GNAS, ANXA4, BPHL, SLC5A2, SLC25A11, GALK1, TAGLN2

SUPPLEMENTARY REFERENCES

1. Aarnisalo AA, Green KM, O'malley J, Makary C, Adams J, Merchant SN, Evans JE: A method for MSE differential proteomic analysis of archival formalin-fixed celloidin-embedded human inner ear tissue. Hearing research 2010;270:15-20.
2. Huang DW, Sherman BT, Lempicki RA: Systematic and integrative analysis of large gene lists using DAVID bioinformatics resources. Nature protocols 2009;4:44.
3. Cox J, Mann M: MaxQuant enables high peptide identification rates, individualized ppb-range mass accuracies and proteome-wide protein quantification. Nature biotechnology 2008;26:1367-1372.
4. Kanehisa M, Goto S: KEGG: kyoto encyclopedia of genes and genomes. Nucleic acids research 2000;28:27-30.

5. Shi J, Walker MG: Gene set enrichment analysis (GSEA) for interpreting gene expression profiles. Current Bioinformatics 2007;2:133-137.
6. Ashburner M, Ball CA, Blake JA, Botstein D, Butler H, Cherry JM, Davis AP, Dolinski K, Dwight SS, Eppig JT: Gene Ontology: tool for the unification of biology. Nature genetics 2000;25:25-29.
